# A systematic review and critical evaluation of inflammatory cytokine associations in hidradenitis suppurativa

**DOI:** 10.12688/f1000research.17267.1

**Published:** 2018-12-13

**Authors:** John W. Frew, Jason E. Hawkes, James G. Krueger

**Affiliations:** 1 Laboratory for Investigative Dermatology, Rockefeller University, New York, NY, 10065, USA

**Keywords:** Hidradenitis Suppurativa, Cytokines, Inflammation, Pathogenesis, IL-17, TNF-alpha

## Abstract

**Background: **The pathogenesis of hidradenitis suppurativa (HS) remains unclear. In order to develop effective treatment strategies, a deeper understanding of pathophysiology is needed. This is impaired by multiple small studies with inconsistent methodologies and the impact of co-occurring pro-inflammatory conditions such as smoking and obesity.

**Methods: **This systematic review aimed to collate all published reports of cytokine studies in tissue, blood, serum and exudate. It was registered with PROSPERO (Registration number CRD42018104664) performed in line with the PRISMA checklist.

**Results: **19 studies were identified comprising 564 individual HS patients and 198 control patients examining 81 discrete cytokines. Methodology was highly varied and the quality of studies was generally low. There was a large degree of variance between the measured levels of cytokines. 78.2% of cytokines demonstrated heterogeneity by the chi-squared test for homogeneity and hence meta-analysis was not deemed appropriate. However, a strong and significant IL-17 signalling component was identified.

**Conclusions: **Cytokines consistently elevated in lesional, peri-lesional and unaffected tissue are identified and discussed. Areas for further investigation include the role of dendritic cells in HS; the contribution of obesity, smoking, diabetes and the microbiome to cytokine profiles in HS; and examining the natural history of this disease through longitudinal measurements of cytokines over time.

## Introduction

Hidradenitis Suppurativa (HS) is a chronic inflammatory disease, the exact pathophysiology of which remains poorly defined ^1^. Dysregulation of the T _h_17: Treg axis ^2^, IL-36 signalling pathways ^3^ and keratinocyte-mediated inflammatory cytokines ^4^ have been demonstrated in lesional skin, blood, serum, and exudate ^5– 8^ although contradictory results exist ^4, 9^. Given the variable and incomplete response of patients to treatment, including monoclonal antibodies ^1^, some authors have proposed clinical ^10, 11^, and immunological ^5^ subtypes of HS in an effort to better predict treatment outcome and response. Thus far, no current schema accurately predicts treatment efficacy.

In order to develop and implement effective treatment strategies in HS, a deeper understanding of the underlying inflammatory pathophysiology is needed. However, due to the heterogeneity of sampling methods, laboratory processing methods and data analysis, comparison across studies is problematic and potentially biased or inaccruate ^12^. Heterogeneity of tissue sampling and laboratory techniques alone may explain the inconsistent and conflicting results regarding specific cytokines, ^4, 9^ however, no systematic analysis of cytokine studies has been undertaken to compare results, methodology, and analytical techniques.

An additional complicating factor is that clinical comorbidities, which are strongly associated with disease activity in HS, such as obesity ^13^, diabetes ^14^, inflammatory bowel disease ^15^, and smoking ^16^, also produce pro-inflammatory cytokines, which affect multiple organ systems including the skin ^15, 17– 19^. Hence, it remains unclear whether the presence or absence of these conditions confound the findings of cytokine studies in HS, and whether clinical stratification of patients is necessary to identify significant pathogenic pathways, which may be amenable to pharmacological intervention. Critical evaluation and analysis of existing studies may also enable meta-analysis, which may identify cytokines, which, in smaller studies, do not have sufficient power to meet statistical significance when compared to controls.

## Objectives

The objectives of this systematic review are:

1) To collate and describe all published reports of human cytokine studies in HS including those in skin, blood, serum and exudate.
2) To critically evaluate the sampling, laboratory and analysis techniques used in each study to assess whether comparisons can be made across individual studies.
3) To analyze the heterogeneity of published studies enable meta-analysis

## Methods

This systematic review was registered with PROSPERO ^20^ (Registration number CRD42018104664) and was conducted in line with the PRISMA checklist ^21^

### Data sources

**Figure 1.**
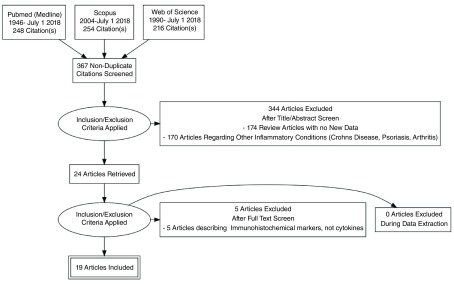
PRISMA Flowchart.

Information sources for this review included PubMed (1946-July 1 2018), Scopus (2004- July 1 2018) and Web of Science (1990-July 1 2018) as shown in Figure 1. Search strategy is presented in Table 1

**Table 1.** Search Strategy.

Resources:	
	1) Pubmed (1946-July 1 2018), 2) Scopus (2004- July 1 2018) 3) Web of Science (1990-July 1 2018) 4) Published Abstracts 5) Contact with Authors for abstracts without full text for clarification of data and methodology
Pubmed Search Strategy:	
	acne inversa OR apocrine acne OR apocrinitis OR Fox-den disease OR hidradenitis axillaris OR HS OR pyodermia sinifica fistulans OR Velpeau’s disease OR Verneuil’s disease OR Hidradenitidis Suppurative AND Cytokine OR chemokine OR inflammatory mediator

### Study eligibility criteria

Eligibility criteria for this review included cohort studies, case-control studies and other observational studies with no restrictions of patient age, sex, ethnicity or language of publication. Eligible studies included:

1) Studies reporting the results of cytokine investigations (in cutaneous tissue, serum, blood or exudate) in human subjects clinically diagnosed with hidradenitis suppurativa.

Studies deemed not eligible included those which:

1) Provide no new data but a review or summary of previously published data
2) Provide no comparison with controls or non-lesional tissue

### Appraisal and synthesis methods

Data collection was performed independently by 2 authors (JWF & JEH), with any disagreements regarding inclusion of citations being referred to a third author (JGK) for mediation. Information was collected using a standardized data collection form (available as Extended data ^22^) with the principal outcomes of interest being the cytokine of interest, measured level of cytokine in lesional HS skin or serum. Comparison data against either peri-lesional, unaffected or control skin or serum was also collated. If data from individual patients was not available then the aggregate data including average change and statistical analyses of the significance of change was collected.

For each individual cytokine, where more than one study reported results, heterogeneity was assessed using the chi-squared tests for homogeneity. Homogeneity was defined as a chi squared value >0.05. All statistical analysis was undertaken using R (version 3.5.1)

Potential sources of bias in the identified studies are acknowledged including the small size of patient cohorts, the variability in sampling, laboratory techniques and the inclusion of patients being treated with a wide-variety of medications including immunosuppressants. Bias was also assessed using the NIH quality assessment tool for observational studies ^23^.

## Results

A total of 367 non-duplicated citations were identified in the literature review ( Figure 1). 343 of these articles were removed upon review of titles and abstracts against the pre-defined eligibility criteria. Full text review of the remaining 24 articles excluded 5 review articles providing no new data. The remaining 19 studies ^2– 9, 24– 33^ included the results of 564 individual HS patients and 198 control patients, which were included in this systematic review.

### Demographics

The summarized demographic data of the patients and controls comprising this review are included in Table 2. The 564 reported cases comprised of 231 males (40.9% reported cases) and 333 females (59.0%). 24 cases were unreported (4.1%). The average age was 38.5 years (n=560, 18 cases unreported). 141 individuals were current smokers (82.4% reported cases), 8 ex-smokers (4.7% reported cases), 22 non-smokers (12.8% reported cases) and 407 unreported. Obesity (BMI>30) was reported in 85 individuals (42.5% reported cases), with 115 (57.5%) individuals non-obese (BMI<30) and unreported in 378 cases. 8 cases reported diabetes mellitus out of 24 reports (33% of reported cases). 12/38 cases reported a positive family history of HS (31.6% reported cases). Hurley Stage was reported as stage 1 in 68 individuals (17.4% reported), stage 2 in 199 individuals (51% reported cases) and stage 3 in 123 individuals (31.6% reported cases) with 188 cases going unreported. The average mHSS (modified hidradenitis suppurativa score) was 78.1 (n=247 cases). Biopsies were largely taken from the axillae (n=32, 43.8%) and groin (n=35, 48.0%), with a minority of samples being taken from the genital and perianal region (n=6, 8.2%). At the time of sampling patients were on treatment including Clindamycin+ Rifampicin (n=18); adalimumab (n=26); Metformin (n=2); levothyroxine (n=1); MABp1 (n=10); tetracyclines (n=12) Infliximab (n=2); other antibiotics (n=4). Treatment was not specified in 74 cases, with no treatment in 86 individuals and treatment withheld in 85 patients.

**Table 2.** Demographic data of included studies.

Number of HS Patients	Male	Female	Mean Age (Years)	Comorbidities				Biopsy Sites			Hurley Staging	mHSS Score (Mean)	Therapy	Study Reference
				Smoking	Obesity (BMI>30)	Diabetes	Family History	Axillae	Groin	Genital				
17		1	45	Ex	Y	NR	NR	Serum Measurements			2	NR	Thyroxine	2
		1	39	Y	N	NR	NR				2	NR	N	
		1	24	N	N	NR	NR				2	NR	N	
		1	41	Y	Y	NR	NR				2	NR	N	
		1	23	Ex	Y	NR	NR				1	NR	N	
		1	35	Y	Y	NR	NR				2	NR	N	
		1	30	Y	N	NR	NR				2	NR	Metformin	
		1	41	Y	Y	NR	NR				3	NR	Clindamycin, Rifampicin	
		1	35	Y	Y	NR	NR				3	NR	Metformin	
		1	47	Y	N	NR	NR				3	NR	N	
	1		19	N	N	NR	NR				1	NR	N	
	1		34	Y	N	NR	NR				2	NR	Adalimumab	
		1	47	N	N	NR	NR				3	NR	Adalimumab, Doxycycline	
		1	32	Y	N	NR	NR				2	NR	Adalimumab	
		1	38	Y	N	NR	NR				3	NR	Adalimumab, Doxycycline	
		1	24	Y	Y	NR	NR				2	NR	Adalimumab	
		1	26	E	Y	NR	NR				2	NR	Adalimumab	
18	11	7	(Range 19–62)	NR	NR	NR	NR				NR	NR	NR	24
15	6	9	38.7	NR	NR	NR	NR	N=9	N=4	N=2	Stage 1=0 Stage 2=10 Stage 3=5		N	3
18	1	1	38	N	Y	NR	N	NR	NR	NR	3	54	N	4
	1		42	Y	N	NR	N	NR	NR	NR	3	56	N	
	1		30	N	Y	NR	Y	NR	NR	NR	3	57	Tetracycline	
		1	43	Y	N	NR	N	NR	NR	NR	1	11	Tetracycline	
		1	32	N	Y	NR	Y	NR	NR	NR	1	14	Tetracycline	
		1	14	N	N	NR	N	NR	NR	NR	3	65	Rifampicin, Clindamycin	
		1	47	Y	N	NR	N	NR	NR	NR	3	44	Tetracycline	
		1	43	Y	Y	NR	N	NR	NR	NR	3	22	N	
		1	21	Y	N	NR	N	NR	NR	NR	1	13	Tetracycline	
		1	47	N	N	NR	N	NR	NR	NR	1	11	Tetracycline	
		1	27	Y	N	NR	N	NR	NR	NR	2	7	Tetracycline	
		1	22	N	N	NR	Y	NR	NR	NR	3	68	N	
	1		50	Y	N	NR	Y	NR	NR	NR	2	46	N	
		1	23	N	N	NR	Y	NR	NR	NR	2	22	N	
		1	19	Y	Y	NR	N	NR	NR	NR	2	26	N	
		1	44	Y	N	NR	Y	NR	NR	NR	2	14	N	
	1		22	Y	N	NR	N	NR	NR	NR	3	23	N	
		1	20	N	N	NR	Y	NR	NR	NR	2	21	Tetracycline	
		1	48	Y	N	NR	N		1		3	NR	Rifampicin, Clindamycin	
	1		25	Y	N	NR	N	1			2	NR	Amoxicillin+ Clav Acid	
		1	20	N	N	NR	N	1			2	NR	N	
		1	31	N	Y	NR	N			1	3	NR	Adalimumab	
	1		40	NA	NA	NR	NA				3	NR	N	
		1	46	Y	N	NR	N			1	3	NR	Tetracycline	
		1	26	Y	N	NR	N	1			2	NR	Azithromycin	
		1	36	Y	N	NR	N		1		2	NR	Amoxicillin+ Clav Acid	
		1	29	N	N	NR	y			1	2	NR	Amoxicillin+ Clav Acid	
24	8	16	36.5 (Range 21–51)	NR	NR	NR	NR	NR	NR	NR	Mean=2.29 (SD=0.62)	NR	Untreated	7
74	36	38	37.4 (SD=12.0)	NR	N=32 (43.2%)	NR	NR	Serum Measurements			Stage 1= 11 Stage 2=47 Stage 3=16		All on treatment (Not further elaborated)	8
8	4	4	41.61 (SD=13.81)	N=5 Y=2 Ex=1	NR	N=4	NR	Exudate Measurements			Stage 1=0 Stage 2=3 Stage 3=5	68.88 (SD=41.45)	NR	6
19 19	11	8	45.6 (SD=10.7)	N=14 (74%)	N=13 (68.4%)	NR	NR	Serum Measurements			Stage 1=0 Stage 2=9 Stage 3=10	82.79 (SD 41.0)	NR	25
												34.5 (SD 43.5)	Adalimumab	
120	43	77	37.3 (SD=5.9)	NR	NR	NR	NR	Serum Measurements			Stage 1=39 Stage2=52.4 Stage 3=44	28.1 (SD=20.2) 52.4 (SD=24.9) 129.3 (SD=79.2)	NR	5
44	13	31	39.1 (SD=11.4)	Y=34 Ex=4	N=16	NR	NR	NR	NR	NR	Stage 1=5 Stage 2=27 Stage 3=12	NR	N=15 Rifampicin, Clindamycin N=1 Minocycline N=2 Adalimumab n=2 Infliximanb n=24 untreated	31
22	10	12	38.2 (Range 19-60)	NR	NR	NR	NR	NR	NR	NR	NR	NR	NR	30
3		1	54	NR	NR	NR	NR	NR	NR	NR	NR	NR	NR	9
	1		36	NR	NR	NR	NR	NR	NR	NR	NR	NR	NR	
		1	59	NR	NR	NR	NR	NR	NR	NR	NR	NR	NR	
10	5	5	42 (Range 21–49)	NR	NR	NR	NR	1	1	N	Stage 2 (100%)	NR	Treatment Withheld	32
20	8	12	37.5 (Range 21–51)	N=18	N=10	NR	NR	NR	NR	NR	NR	NR	Treatment Withheld (8 weeks prior)	29
25	9	16	36 (Range 18–51)	NR	NR	NR	NR	NR	NR	NR	Mean =2.16 (SD=0.55)	NR	Treatment Withheld (3 weeks prior)	28
47	19	28	42.3 (Range 22–54)	NR				Serum Measurements				48.3 (Range 8–144)	NR	27
11	9	2	39.6 (Range 18–61)	NR	NR	NR	NR	NR	NR	NR	“Mod-Severe Disease”	NR	NR	
20	6	14	40 (SD=15)	19	27.6 (4.1)	NR	NR	7	12	1	Stage 1=4 Stage 2=11 Stage 3=5		Treatment withheld 3 weeks prior	26
10	1	9	38 (SD=15)	10	28.9 (SD 4.5)	NR	NR	3	7	0	Stage1=2 Stage2=7 Stage3=1		Treatment Withheld 3 weeks prior	
10	7	3	46.6 (SD=15.1)	10	29.4 (4.7)	3	2	Serum			Stage 3=10	195.6 (SD=97.9)	MABp1	33
10	6	4	49.3 (SD=9.8)	8	27.9 (7.1)	1	2				Stage 2=2 Stage 3=8	124.9 (SD=73.7)	No Treatment	
TOTAL: 564	231	333	38.5	141	85 (0f 200)	8 (of 24)	12	32	35	6	Stage 1= 68 Stage 2=199 Stage 3=123	Average =78.1 (n=247)	Clindamycin+ Rifampicin=18; Adalimumab=26; Metformin=2; Treatment withheld= 85; Thyroxine=1; MABp1=10; Tetracycylines=12; No Treatment=86; Not Specified=74; Infliximab=2; Antibiotics=4; Not Reported=258	

BMI= Body Mass Index mHSS= modified Hidradenitis Suppurativa Score (Sartorius Score) NR= Not Reported SD= Standard Deviation Y= Yes N=No Ex= Ex Smoker

Only 5/19 (26.3%) studies analysed both lesional tissue and serum levels of cytokines, enabling direct comparison between these two compartments. 8/19 (42.1%) studies provided age and sex matched controls, 5/15 (33.3%) studies stratified by disease severity and no studies stratified by lesion site or comorbidities. 8/19 (42.1%) studies stratified or accounted for treatment or reported discontinuing treatment up to 3 weeks prior to sample collection ( Table 3).

**Table 3.** Critical evaluation of methodology of studies included in this review.

Cytokines Measured	Number of HS Patients	Number of Controls	Samples Analyzed	Age/Sex Matched Controls	Timing of Samples	Stratified by severity	Stratified by lesion site	Stratified by Co- morbidities	Stratified by Treatment	Sample Storage Time	Sample Types	Study Reference
IL-17 IL-22 IFNg IL-2 IL-10 GM-CSF	17	9	L, PL, U, C, S	Y	NR	NR	N	N	Y	NR	Skin, Serum	2
S100A7 Lysozyme LL37 hBD3 α-MSH MIF TNF-α IL-8 MHC1	18	12	L	N	NR	NR	N	N	N	NR	Skin	24
IL-36α IL-36β IL-36g	15	15	L, PL	NR	NR	NR	N	N	N	NR	Skin	3
IL-17 IL-22 IFNg CCl20 CCL27 S100A7 S100A8 IL-1B CCL5 IP10 IL-8 IL-6 TNF-α	18	18	L, PL, S	Y	NR	Y	N	N	N	NR	Skin, Serum	4
LL37 IL-17 TNF-α IL-23 IL-1b IL-10 IL-32	24	9	L	Y	NR	NR	NR	N	Y (untreated)	NR	Skin	7
IL-6 IL-23 TNF-α R1 IL-1β IL-8 IL-10 IL-12p70 IL17A TNFR2 CRP ESR	74	22	Serum only	N	NR	Y	NR	N	N	NR	Serum	8
IFNg, IL-12p70,IL-1β IL-1α IL-17A IL-6 TNF-α TNF-β IL-16 IL-12/23p40 IL-10 IL-4 IL-13 IL-2 IL-15 IL-7 IL-5 GM-CSF VEGF	8	8	Wound Exudate	Y	NR	N	N	N	N	NR	Wound Exudate	6
IL-1B IL-6 IL-8 IL-10 IL-17A IL-23 TNFR1 TNFR2	19	19	Serum only	N	Y (Fasting)	N	N	N	Y (Adalimumab)	NR	Serum only	25
TNF-α, IL-1B, IL-6 IL-10 IL-17 IL-22 IL-1RA	120	24	Serum and Pus	Y	N	Y	N	N	Y (Etanercept)	NR	Serum Pus	5
IL-17 IL-1B IL-10 TNF-α	44	5	L, PL, U	N	N	N	N	N	N	NR	Skin	31
IL-17 Caspase1 NLRP3 S100A8 S100A9	22	Yes (NR)	L, PL, U, C	NR	NR	N	N	N	N	NR	Skin	30
TNF-α IL-1β IL-6 IFNg IL-17A IL-22	3	(Unknown)	S	Y	NR	N	N	N	N	NR	Serum	9
IL1-2p70 IL-23p19 IL-17	10	8	L, C	N	NR	N	N	N	Y (ceased 3/25 prior)	NR	Skin	32
IL-32 IL-32α IL-32β IL-32d IL-32g IFNg IL-17 IL-13	20	10	L, C, S	N	NR	Y	N	N	Y (ceased 8/52 prior)	NR	Skin, Serum	29
IL-36α IL-36β IL-36g IL-36RA	25	7	L, C, S	N	NR	N	N	N	Y (ceased 3/25 prior)	NR	Skin Serum	28
TNF-α IFNg IL-1β IL-6 IL-10 IL-19, IL-17A IL-22 IL-36b IL-12/23p40 IL-22 E Selectin P Selectin CXCL6 CXCL11 CX3CL1 CCL2 CCL18 CXCL9 sVEGFR1 MMP2 Cystatin C LCN2	10	16	L	Y	NR	N	N	N	N	NR	Skin Serum	27
IL-1β IL-2 IL-4 IL-5 IL-6 IL-8 IL-10 IL- 12p70 TNF-α IFNg	20	6	L, PL, C	N	NR	Y	N	N	N	NR	Skin	26
IL-1α, IL-8	10	10	S	N	NR	N	N	N	Y	NR	Serum	33

Table 2: Critical Evaluation of Methodology of Studies Included in This Review Key:L= Lesional, PL= Perilesional, U= Uninvolved, C= Control S=Serum, Y=Yes, N=No, NR= Not Reported,

### Cytokine analysis

A total of 81 discrete cytokines were analysed over the 19 studies (presented in Table 4). 6 studies provided a total of 78 outcomes from tissue of lesional or peri-lesional biopsies, 4 studies provided a total of 30 results from serum analysis and 1 study provided 15 results from exudate analysis. The remaining 8 studies did not provide quantification of cytokine levels but did provide analysis of the change and significance between lesion and control samples. The degree of change between lesional and control samples varied widely from 1.5 times the control level (IL-1RA p=0.0112) to 149 times the control level (IL-17 p<0.05). 33 cytokines were evaluated in more than one study. Only IL-1β, IL-6, IL-8, IL-17A and TNF-α had data from 5 or more separate studies.

**Table 4.** Reported cytokine results of studies included in this systematic review.

Target Cytokine	Mean Level in Patient Serum (pg/mL)	Mean Level in Control Serum (pg/mL)	Mean Level in Lesional Tissue (pg/mL)		Mean Level in Perilesional Tissue (pg/mL)	Mean Uninvolved Tissue Levels (pg/mL)	Mean Control Tissue Levels (pg/mL)	Fold Increase	Comparison and Significance		Comparison and Significance		Study Reference
IL-1α			1126				2549		Le:Ce	P= 0.53			6
			0.2				0.1	NR	L:C	NS			26
	772.0	697.2							HSs:Cs	NS			33
IL-1RA			44.0				29.6	1.5	L:C	P= 0.0112			26
IL-1β	0.9	0.4							HSs:Cs	P=0.801			8
			862.5				1503		Le:Ce	P= 0.69			6
									L:C	NS	Lpa:C	NS	25
			SERUM ONLY						HSs:Cs	P= 0.044			5
			100		10	3	1	115 fold	L:C	P= 0.001	PL:C	0.05	31
									L:U	P= 0.01	U:C	NS	
								R=0.7 ^#^	L:C	NS			7
			1.6				0.0	54.4	L:C	P= 0.0028			26
IL-4			6.56				9.77		Le:Ce	P= 0.54			6
			0.0				0.1		L:C	NS			7
IL-5			0.2				0.2		L:C	NS			7
			30.15				9.314		Le:Ce	P= 0.17			6
IL-6									L:C * L:C ** L:C ***	NS NS NS			4
	6.2	0.6							HSs:Cs	P= 0.001			8
			2377				5451		Le:Ce	NS			6
									L:C	P= 0.05	Lpa:C	0.05	25
			SERUM ONLY						HSs: Cs ^+++^	P= 0.002			5
			124.4			101.9			L:C	NS			7
sIL-6R			16.3				4.4	3.7	L:C	P= 0.0028			7
IL-8	NR	NR	i69.6 / s67.6				64.9		Li:C	P<0.01	Ls:C	P<0.001	24
									L:C * L:C ** L:C ***	NS NS NS			4
	27.9	36.3							HSs:Cs	NS			8
									L:C	P= 0.05	Lpa:C	NS	25
			1401				12.0		L:C	NS			7
	1000	3000							L:C	P= 0.049			33
IL-10									L:C	P<0.05			4
	3.4	3.3							HSs:Cs	NS			8
			19.85				34.74		Le:Ce	NS			6
									L:C	P= 0.05	Lpa:C	0.05	25
			SERUM ONLY						HSs:Cs ^+^	P= 0.0001			5
			SERUM ONLY						HSs:Cs ^++^	P= 0.0001			5
			3.8		1.1		0.4	3-4	L:C	P= 0.01	PL:C	NS	31
									L:U	P= 0.01	U:C	NR	
	3	2							HSs:Cs	NS			27
			19.2				1.3	14.8	L:C	P= 0.0028			7
IL-11			78.6				7.2	11.0	L:C	P= 0.0056			7
IL-12p40			488.3				97.86		Le:Ce	P= 0.07			6
	75	75							HSs:Cs	NS			27
			0.5				0.4		L:C	NS			7
IL-12p70	3.4	0.6							HSs:Cs	P= 0.427			8
			9.412				15.02		Le:Ce	P= 0.609			6
			0.0				0.0		L:C	NS			7
IL-13			70.98				55.61		Le:Ce	P= 0.56			6
			0.0				0.1		L:C	NS			7
IL-15			24.5				5.61		Le:Ce	P= 0.18			6
			1.9				2.9		L:C	NS			7
IL-16			15277				15586		Le:Ce	P= 0.97			6
			22.3				4.2	5.3	L:C	P= 0.0028			7
IL-17									S:C	P<0.005			4
					SERUM ONLY		SERUM ONLY	SERUM ONLY		HSs:Cs ^+^	0.014		5
					SERUM ONLY		SERUM ONLY	SERUM ONLY		HSs:Cs ^++^	0.005		5
			150		45	1	1	149 fold	L:C	P= 0.05	PL:C	0.05	31
									L:PL	NS	U:C	0.05	
						No Quantification			L:C	↑(NS)	L:PL	No Diff	30
								R=0.66 ^#^		NS			27
IL-17A									L:C	P<0.005			4
	5.6	0.3							HSs:Cs	NS			8
			1006				32.7		Le:Ce	NS			6
									L:C	P= 0.05	Lpa:C	NS	25
	4	5							HSs:Cs	NS			27
			8.1		NR		1.1	7.3	L:C	P= 0.0056			26
IL-22									L:C	NS			4
	8.8	0.0							HSs:Cs	NS			8
IL-23									L:C	NS	Lpa:C	0.05	25
								R=0.68 ^#^		NS			7
IL-32	50ng/mL	1ng/mL	Only Normalised Values Provided					4 (skin) 50 (serum)	L:C	P= 0.01	HSs: Cs	p<0.05	29
IL-32α								3 fold	L:C	P= 0.01			29
IL-32β								2 fold	L:C	P= 0.05			29
IL-32g								Not elevated	L:C	P= 0.001			29
IL-32d								3 fold	L:C	NS			29
IL-36α			0.4		0.02		0.02		L:C	P=0.0174	PL:C	NS	3
	250	0					1	45.07 fold	L:C	P= 0.01			28
IL-36b			4.33		3.00		0.51		L:C	P= 0.0001	PL:C	0.0035	3
	15	4					1	1.45 fold	L:C	P= 0.25			28
IL-36g			3.64		0.83		0.49		L:C	P= 0.0161	PL:L	0.0302	3
	100	20					1	1.96 fold	L:C	P= 0.07			28
IL-36RA			0.46		0.28		0.06		L:C	P= 0.0001	PL:C	0.0003	3
	50	100	No Quantificaiton					No Increase	L:C	P= 0.10			28
IL-37			3.24		14.7		1.81		PL:L	P= 0.0002	PL:C	0.0001	3
IL-38			0.09		0.19		0.06		L:C	P= 0.0230	PL:C	0.0069	3
TNF-α			i69.4	66.6 s	NR		65.8	NR	Li:C	NS	Ls:C	NS	24
									L:C * L:C ** L:C ***	NS NS NS			4
					83.26			65.74	Le:Ce	P= 0.7			6
			SERUM ONLY		SERUM ONLY				HSs:Cs ^+^	P=0.021			5
			2.2		1.3	0.6	0.7		L:C	P=0.01	PL:C	0.01	31
									L:PL	NS	U:C	NS	
			0.3				0.2	1.6	L:C	P=0.0336			26
TNF-β			9.24				1.65		Le:Ce	P=0.03			6
			0.4				0.4	NR	L:C	NS			26
sTNFR1	879.8	325.9							HSs:Cs	P <0.001			8
									L:C	NS	Lpa:C	0.05	25
			78.0				40.2	1.9	L:C	P= 0.0112			26
sTNFR2	927.9	527.4							HSs:Cs	P= 0.053			8
									L:C	P= 0.05	Lpa:C	0.05	25
			47.0				8.1	5.8	L:C	P= 0.0028			26
hBD1			0.019 0.021 0.018				0.058 0.077 0.095	0.3 0.3 0.2	L:C * L:C ** L:C ***	P= 0.240 P= 0.132 P= 0.026			4
hBD2			0.013 0.019 0.058				0.011 0.018 0.067	1.1 1.1 0.9	L:C * >L:C ** L:C ***	P= 0.937 P= 0.699 P= 0.937			4
hBD3			76.9 i	75.7 s			72.5	NR	Li:C	P<0.05	Ls:C	NS	24
			0.33 0.33 0.379				0.117 0.125 0.203	2.8 2.6 1.9	L:C * L:C ** L:C ***	P= 0.485 P= 0.394 P= 0.485			4
S100A7			i84.8	77.8 s			71.5	NR	Li:C	P<0.001	Ls:C	P<0.05	24
			1.516 1.625 2.297				0.177 0.354 0.707	8.6 4.6 3.2	L:C * L:C ** L:C ***	P= 0.009 P= 0.180 P= 0.132			4
S100A8			24.251 25.992 24.251				4.925 11.314 10.556	4.9 2.3 2.3	L:C * L:C ** L:C ***	P= 0.240 P= 0.537 P= 0.393			4
								NR	L:C	↑ (NS)	L:PL	↑ (NS)	30
S100A9			0.003 0.005 0.003				0.002 0.004 0.006	1.7 1.1 0.6	L:C * L:C ** L:C ***	NS NS NS			4
									L:C	↑ (NS)	L:PL	↑ (NS)	30
LL37			84.1 i /80.9 s				75.8		Li:C	P<0.05	Ls:C	NS	24
Lyzozyme			55.2 i / 52.7 s				59.6		Li:C	NS	Ls:C	P<0.05	24
MIF			77.8 i/ 77.8 s				70.7		Li:C	NS	Ls:C	P<0.01	24
αMSH			NR		i74.6 i / 73.1 s		NR	70.9	Li:C	P<0.01	Ls:C	P<0.01	24
MHC1			75.5 i/74.7 s				74.4		Li:C	NS	Ls:C	NS	24
RNase7			0.435 0.330 0.574				0.063 0.077 0.109	7.0 4.3 5.3	L:C * L:C ** L:C ***	P= 0.145 P= 0.589 P= 0.179			4
IP10			89.9				12.6		L:C * L:C ** L:C ***	P<0.05 P<0.005 P<0.05			4
CCL3			0.4				0.2	2.0	L:C	P= 0.0196			26
CCL5			- 46.1 -				- 6.2 -		L:C * L:C ** L:C ***	P<0.05 P<0.05 NS			4
			7.6				1.4	5.4	L:C	P= 0.0112			26
CCL20									L:C	P<0.005			4
CCL27									L:C	P<0.05			4
CRP	13.4	1.2							HSs:Cs	p<0.001			8
									L:C	P= 0.05	Lpa:C	0.05	25
ESR	29.5	10.2							HSs:Cs	<0.001			8
									L:C	P= 0.05	Lpa:C	0.05	25
IFNg								R=0.7	L:C	NS			7
								<5% Normal	HSs:Cs	↑ (NS)			9
			1418				102.5		Le:Ce	P= 0.027			6
									HSs:Cs	P<0.05	L:C	P<0.05	4
GMCSF			78.45				82.13		Le:Ce	P= 0.96			6
			0.4				0.0	NR	L:C	NS			26
VEGF			632.1				1544		Le:Ce	P= 0.23			6
sVEGFR1	60	60							HSs:Cs	NS			27
Caspase 1							No Quanti	No Quanti	L:C	↑ (NS)	L:PL	↑ (NS)	30
NLRP3							No Quanti	No Quanti	L:C	↑ (NS)	L:PL	NS	30
CAMP								4	L:C	NS			7
Uteroglobulin	20	20							HSs:Cs	NS			27
Cystatin C	0.85	0.8							HSs:Cs				27
LCN2	90	40	0.5			0.02			HSs:Cs	<0.001	L:C	<0.001	27
BD2	0.9	1							HSs:Cs	NS			27
MMP2	200	210							HSs:Cs	<0.05			27
BLC			8.1				0.58	10.5	L:C	P= 0.0056			26
ICAM-1			98.7				31.9	3.1	L:C	P= 0.0028			26
Eotaxin			0.1				0.1	NR	L:C	NS			26
Eotaxin2			3.9				2.5	NR	L:C	NS			26
CXCL6	160	140								NS			27
CXCL9			219.8				13.8	16	L:C	P= 0.0028			26
CXCL11	0.4	0.4								NS			27
CX3CL1	0.9	1								NS			27
I-309			0.4				0.3	NR	L:C	NS			26
MCP1			47.5				37.1	NR	L:C	NS			26
M-CSF			0.4				0.2	NR	L:C	NS			26
MIP1b			16.1				5.8	NR	L:C	NS			26
MIP1d			0.1				0.1	NR	L:C	NS			26
PDGF			0.5				0.2	NR	L:C	NS			26
TIMP1			260.1				166.2	NR	L:C	NS			26
TIMP2			989.2				997.3	NR	L:C	NS			26

Key: L= Lesional ; PL= Perilesional; C= Control; NS= Not Significant ; HSs= HS Serum; Cs= Control Serum; HSe= HS Exudate; Ce= Control Exudate; I = Inflamed lesional skin, S= Scarred lesional skin, #= Vs CAMP, *= NT (Non-Treated) Samples ,** = Stimulation by Pam2CSK4 Lipopeptide,*** Stimulation by Muramyl Dipeptide (MDP), + Heat Killed Candida Albicans; ++ Heat Killed Staph Aureus, +++ Lipopolysaccharide;

Cytokines and inflammatory proteins which were elevated in more than one study in lesional tissue included IL-1β, IL-6R, IL-10, IL-17A, IL-36α, IL-36β, IL-36 **γ**, IL-36RA, TNF-α, sTNFR2, hBD1, hBD2, hBD3, s100A7, LL37/Cathelicidin, CCL3, CCL5, CCL27 and BLC. Cytokines and inflammatory proteins elevated in peri-lesional tissue included IL-1β, IL-17, IL-36β, IL-36RA, IL-37, IL-38 and TNF-α. IL-37 was the only cytokine identified which showed significant differences between lesional and peri-lesional tissue, with a 1.81 times elevation in lesional compared to peri-lesional tissue (p=0.0002) ^3^. IL-17 was elevated in unaffected HS tissue compared to control patient tissue (p<0.05) in one study ^31^. In HS tissue, S100A9, hBD1 and hBD2 were reduced but this data did not meet statistical significance. Two studies measuring IL-1β levels showed no statistically significant difference between lesional and control skin ^7, 25^. No significant elevation of IL-6 was seen in lesional tissue compared to control with the exception of 1 study ^25^. IL-8 levels only just made significance in two studies ^5, 7^, with one study showing significant elevation of IL-8 in lesional compared to control tissue ^24^. Two additional studies showed no significant difference ^4, 8^. TNF-α levels were significantly elevated compared to control tissue in two studies ^7, 31^ but not significantly in 2 additional studies ^4, 24^. sTNFR1 was significantly elevated in one study ^26^ whilst showing a non-significant difference in a second study ^25^. CCL5 was significant in 2 studies in lesional tissue compared with controls ^4, 26^. One methodology using muramyl dipeptide (MDP) did not reach statistical significance compared to stimulation with Pam2CSK4 Lipopeptide, and non-treated (NT) cells. IFN- **γ** was elevated in lesional tissue with no significance in one study ^28^ and significance in another ^4^.

Elevated cytokines and inflammatory proteins in HS serum included IL-1β, IL-6, IL-8, IL-10, IL-12p70, IL-17, TNF-α, sTNFR1, CRP, ESR, LC2, and MMP2. TNF-β, and IFN-γ were elevated in wound exudate from active HS lesions. IFN-γ was noted to be decreased in HS patient serum compared to healthy control serum, despite the elevation in wound exudate. Conflicting results were seen in serum findings in IL-10, IL-17 and IFN-γ. One study demonstrated elevated serum IL-10 levels compared to control ^5^ whereas two other studies ^8, 27^ showed no significant difference. Whilst two studies ^4, 5^ illustrated elevated IL-17 Serum levels in HS patients, one study ^7^ showed no significant difference between patients and controls. IFN-γ showed no statistically significant decrease in the serum of HS patients compared to control in one study ^9^ but a significant difference in a larger, higher powered study ^4^.

Because adalimumab improves HS through TNF antagonism ^1, 2^, this cytokine must be classified as pathogenic. TNF mediates inflammation in a classic “sepsis” cascade in tissues—in this pathway LPS from gram negative bacteria activates TNF release from cells, and then TNF stimulates production of IL-1b, IL-6, and IL-8, leading to neutrophil attraction into sites of infection ^2, 4^. Increases in IL-1β and IL-8 measured in HS, as well as neutrophil accumulation, could result from this pathway. Alternatively, in psoriasis, TNF is a major cytokine that acts on the IL-23/Type 17 T-cell pathway at two points. First TNF induces IL-23 synthesis in myeloid (CD11c+) dendritic cells in the skin ^34^. Second, TNF (as well as other cytokines that also activate NF-kB) act synergistically with IL-17A or IL-17F to increase synthesis of many other cytokines, chemokines, and inflammatory molecules in keratinocytes and other cell types. There are several clues that an IL-23/Type17 T-cell pathway may be active in HS which include detection of T _h_17 T-cells in skin infiltrates, increased production of IL-17A, and increased production of LL-37/cathlecidin, S100A7, S100A8, S100A9, LCN2, IL-8, beta-defensins and IL-36; which are all molecules induced by IL-17 in keratinocytes, as also the presence of psoriasis-like epidermal hyperplasia in some reports. The increased production of CCL20 ^4^, would be predicted to increase tissue infiltration of both T _h_17 T-cells and CD11c+ DCs, which have both been observed in HS, and increased production of TGF-β could increase differentiation of T _h_17 T-cells from precursors and/or influence scarring in skin lesions. If IL-17 is driving inflammation in HS, one would expect to see increased production of additional chemokines that regulate neutrophil chemoattraction (CXCL1, CXCL2, CXCL3). Epidermal hyperplasia is not presently explained in HS, but this could be related potentially to increased expression of IL-19, IL-20 or IL-22, which are associated with the IL-23/Type 17 T-cell axis. If IL-22 is produced in HS lesions, this would implicate T _h_22 T-cells as a T-cell type also associated with the IL-23/Type 17 T-cell axis. There is an uncertain role for other T-cell subsets in HS. Increased production of CXCL9 and IP-10 (CXCL10) are often linked to production of IFN-γ from T _h_1 T-cells in inflammatory sites, but IL-26 or IL-29, which are also cytokines produced by T _h_17 T-cells are alternative activators of STAT1 and CXCL9 production. IL-32 production in HS may also be linked to a T-cell subset that produces this cytokine. Low production of T _h_2 associated cytokines (IL-4, IL-5, or IL-13) has been measured in HS, suggesting an unlikely role of this T-cell subset. Likewise, the presence and function of T regulatory cells (Tregs) in HS lesions needs further study. IL-10 which is elevated in HS could be produced by either Tregs or the cDC1 (BDCA3+) DC subset, but levels may be inadequate to control tissue inflammation. At present, dendritic cell subsets are also incompletely characterized in HS. Potential sources of IL-12 or IL-23 are CD11c+ DCs, which includes the tissue resident BDCA-1+ (cDC2) subset and less mature inflammatory DCs, which are abundant cells in inflammatory lesions of psoriasis or atopic dermatitis but have not been investigated in HS. Cytokine contributions by other cell types such as innate lymphoid cells, macrophages, mast cells, and other leukocytes also remains to be determined.

### Cytokine analysis methods

The methodologies of cytokine analysis varied widely ( Table 5). 92 results were produced using electrochemical luminescence (ECL) procedures from three separate systems and manufacturers. 62 results were produced using ELISA. 18 results ^4^ were performed with either ELISA or ECL but not further specified. 15 results were produced using polymerase chain reaction (PCR) with three separate systems from three manufacturers. Four discrete cytokines (IL-10, IL-17, TNF-α and IFN-γ) were analysed using all three techniques (ECL, ELISA and PCR), whilst 15 discrete cytokines (IL-6, IL-8, IL12p40, IL-17A, IL-22, IL-23, S100A7, S100A8, S100A9, RNAse7, IP-10, CCL5, CCL20, CCL27) were analysed using ELISA and ECL only. We note IL-17 levels may well be below the lower limit of quantification with ELC and ELISA based approaches, with only the Singulex platform having the ability to quantify levels of IL-17 present in blood and serum of normal subjects.

**Table 5.** Cytokine analysis methodology of studies included in this review.

Cytokine	Method	Details	Study
IL-1α	ECL	Meso Scale Discovery electrochemiluminescent assay (MSD, Meso Scale Diagnostics, Rockville, MD MSD V-Plex cytokine panel 1 and the V-plex proinflammatory panel 1	6
	ECL	(CBA Human Inflammation kit and CBA Human TH1⁄TH2 Cytokine kit; BD Biosciences, Franklin Lakes, NJ, U.S.A.) Analyssi: FACSCalibur (BD Biosciences)	26
IL-1ra	ECL	(CBA Human Inflammation kit and CBA Human TH1⁄TH2 Cytokine kit; BD Biosciences, Franklin Lakes, NJ, U.S.A.) Analyssi: FACSCalibur (BD Biosciences)	26
IL-1β	ECL	xMAP technology (Luminex Corporation, Austin, TX, USA)	8
	ECL	Meso Scale Discovery electrochemiluminescent assay (MSD, Meso Scale Diagnostics, Rockville, MD MSD V-Plex cytokine panel 1 and the V-plex proinflammatory panel 1	6
	ECL	xMAP technology (Luminex Corporation, Austin, TX, USA)	25
	ELISA	Cytokines were measured in duplicate by ELISA (R&D Minneap- olis, USA).	5
	PCR	IL10, IL17A, IL1Β, IL18 and NLRP3 was performed with predesigned Taqman gene expression assays (Applied Biosystems) on a Roche Light Cycler (Roche, Pleasanton, CA, U.S.A.)	31
	PCR	(Hs01555410_m1), ABI-Prism 7300 Sequence Detector System (Applied Biosystems	7
	ECL	(CBA Human Inflammation kit and CBA Human TH1⁄TH2 Cytokine kit; BD Biosciences, Franklin Lakes, NJ, U.S.A.) Analyssi: FACSCalibur (BD Biosciences)	26
IL-4	ECL	Meso Scale Discovery electrochemiluminescent assay (MSD, Meso Scale Diagnostics, Rockville, MD MSD V-Plex cytokine panel 1 and the V-plex proinflammatory panel 1	6
	ECL	(CBA Human Inflammation kit and CBA Human TH1⁄TH2 Cytokine kit; BD Biosciences, Franklin Lakes, NJ, U.S.A.) Analyssi: FACSCalibur (BD Biosciences)	7
IL-5	ECL	(CBA Human Inflammation kit and CBA Human TH1⁄TH2 Cytokine kit; BD Biosciences, Franklin Lakes, NJ, U.S.A.) Analyssi: FACSCalibur (BD Biosciences)	7
	ECL	Meso Scale Discovery electrochemiluminescent assay (MSD, Meso Scale Diagnostics, Rockville, MD MSD V-Plex cytokine panel 1 and the V-plex proinflammatory panel 1	6
IL-6	ELISA/ ECL	ELISA (Quantikine; R&D Systems) or Luminex assay (Millipore, Billerica, MA).	4
	ECL	xMAP technology (Luminex Corporation, Austin, TX, USA)	8
	ECL	Meso Scale Discovery electrochemiluminescent assay (MSD, Meso Scale Diagnostics, Rockville, MD MSD V-Plex cytokine panel 1 and the V-plex proinflammatory panel 1	6
	ECL	xMAP technology (Luminex Corporation, Austin, TX, USA). The Milliplex MAP multiplex assay	25
	ELISA	Cytokines were measured in duplicate by ELISA (R&D Minneap- olis, USA).	5
	ECL	(CBA Human Inflammation kit and CBA Human TH1⁄TH2 Cytokine kit; BD Biosciences, Franklin Lakes, NJ, U.S.A.) Analyssi: FACSCalibur (BD Biosciences)	7
sIL-6R	ECL	(CBA Human Inflammation kit and CBA Human TH1⁄TH2 Cytokine kit; BD Biosciences, Franklin Lakes, NJ, U.S.A.) Analyssi: FACSCalibur (BD Biosciences)	7
IL-8	ELISA	pABG AHC0881 1:50 rabbit antihuman	24
	ELISA/ ECL	ELISA (Quantikine; R&D Systems) or Luminex assay (Millipore, Billerica, MA).	4
	ECL	xMAP technology (Luminex Corporation, Austin, TX, USA)	8
	ECL	xMAP technology (Luminex Corporation, Austin, TX, USA)	25
	ECL	(CBA Human Inflammation kit and CBA Human TH1⁄TH2 Cytokine kit; BD Biosciences, Franklin Lakes, NJ, U.S.A.) Analyssi: FACSCalibur (BD Biosciences)	7
	ELISA	Cytokines were measured in duplicate by ELISA (R&D Minneap- olis, USA).	33
IL-10	ELISA/ ECL	ELISA (Quantikine; R&D Systems) or Luminex assay (Millipore, Billerica, MA).	4
	ECL	xMAP technology (Luminex Corporation, Austin, TX, USA)	8
	ECL	Meso Scale Discovery electrochemiluminescent assay (MSD, Meso Scale Diagnostics, Rockville, MD MSD V-Plex cytokine panel 1 and the V-plex proinflammatory panel 1	6
	ECL	xMAP technology (Luminex Corporation, Austin, TX, USA)	25
	ELISA	Cytokines were measured in duplicate by ELISA (R&D Minneap- olis, USA).	5
	ELISA	Cytokines were measured in duplicate by ELISA (R&D Minneap- olis, USA).	5
	PCR	IL10, IL17A, IL1Β, IL18 and NLRP3 was performed with predesigned Taqman gene expression assays (Applied Biosystems) on a Roche Light Cycler (Roche, Pleasanton, CA, U.S.A.)	31
	ELISA	Quantikine enzyme-linked immunosorbent assay (ELISA) systems from Bio-Techne	27
	ECL	(CBA Human Inflammation kit and CBA Human TH1⁄TH2 Cytokine kit; BD Biosciences, Franklin Lakes, NJ, U.S.A.) Analyssi: FACSCalibur (BD Biosciences)	7
IL-11	ECL	(CBA Human Inflammation kit and CBA Human TH1⁄TH2 Cytokine kit; BD Biosciences, Franklin Lakes, NJ, U.S.A.) Analyssi: FACSCalibur (BD Biosciences)	7
IL-12p40	ECL	Meso Scale Discovery electrochemiluminescent assay (MSD, Meso Scale Diagnostics, Rockville, MD MSD V-Plex cytokine panel 1 and the V-plex proinflammatory panel 1	6
	ELISA	Quantikine enzyme-linked immunosorbent assay (ELISA) systems from Bio-Techne	27
	ECL	(CBA Human Inflammation kit and CBA Human TH1⁄TH2 Cytokine kit; BD Biosciences, Franklin Lakes, NJ, U.S.A.) Analyssi: FACSCalibur (BD Biosciences)	7
IL-12p70	ECL	xMAP technology (Luminex Corporation, Austin, TX, USA)	8
	ECL	Meso Scale Discovery electrochemiluminescent assay (MSD, Meso Scale Diagnostics, Rockville, MD MSD V-Plex cytokine panel 1 and the V-plex proinflammatory panel 1	6
	ECL	(CBA Human Inflammation kit and CBA Human TH1⁄TH2 Cytokine kit; BD Biosciences, Franklin Lakes, NJ, U.S.A.) Analyssi: FACSCalibur (BD Biosciences)	7
IL-13	ECL	Meso Scale Discovery electrochemiluminescent assay (MSD, Meso Scale Diagnostics, Rockville, MD MSD V-Plex cytokine panel 1 and the V-plex proinflammatory panel 1	6
	ECL	(CBA Human Inflammation kit and CBA Human TH1⁄TH2 Cytokine kit; BD Biosciences, Franklin Lakes, NJ, U.S.A.) Analyssi: FACSCalibur (BD Biosciences)	7
IL-15	ECL	Meso Scale Discovery electrochemiluminescent assay (MSD, Meso Scale Diagnostics, Rockville, MD MSD V-Plex cytokine panel 1 and the V-plex proinflammatory panel 1	6
	ECL	(CBA Human Inflammation kit and CBA Human TH1⁄TH2 Cytokine kit; BD Biosciences, Franklin Lakes, NJ, U.S.A.) Analyssi: FACSCalibur (BD Biosciences)	7
IL-16	ECL	Meso Scale Discovery electrochemiluminescent assay (MSD, Meso Scale Diagnostics, Rockville, MD MSD V-Plex cytokine panel 1 and the V-plex proinflammatory panel 1	6
	ECL	(CBA Human Inflammation kit and CBA Human TH1⁄TH2 Cytokine kit; BD Biosciences, Franklin Lakes, NJ, U.S.A.) Analyssi: FACSCalibur (BD Biosciences)	7
IL-17	ELISA/ ECL	ELISA (Quantikine; R&D Systems) or Luminex assay (Millipore, Billerica, MA).	4
	ELISA	Cytokines were measured in duplicate by ELISA (R&D Minneap- olis, USA).	5
	PCR	IL10, IL17A, IL1Β, IL18 and NLRP3 was performed with predesigned Taqman gene expression assays (Applied Biosystems) on a Roche Light Cycler (Roche, Pleasanton, CA, U.S.A.)	31
	PCR	IL-17 (clone AF-317-NA; R&D Systems, Wiesbaden, Germany),	30
	PCR	IL-17 (Hs00174383_m1), ABI-Prism 7300 Sequence Detector System	27
IL-17A	ELISA/ ECL	ELISA (Quantikine; R&D Systems) or Luminex assay (Millipore, Billerica, MA). eBioscience, Paris, France	4
	ECL	xMAP technology (Luminex Corporation, Austin, TX, USA)	8
	ECL	Meso Scale Discovery electrochemiluminescent assay (MSD, Meso Scale Diagnostics, Rockville, MD MSD V-Plex cytokine panel 1 and the V-plex proinflammatory panel 1	6
	ECL	xMAP technology (Luminex Corporation, Austin, TX, USA)	25
	ELISA	Quantikine enzyme-linked immunosorbent assay (ELISA) systems from Bio-Techne	27
	ECL	(CBA Human Inflammation kit and CBA Human TH1⁄TH2 Cytokine kit; BD Biosciences, Franklin Lakes, NJ, U.S.A.) Analyssi: FACSCalibur (BD Biosciences)	26
IL-22	ELISA	ELISA (Quantikine; R&D Systems) or Luminex assay (Millipore, Billerica, MA). eBioscience, Paris, France	4
	ECL	xMAP technology (Luminex Corporation, Austin, TX, USA)	8
IL-23	ECL	xMAP technology (Luminex Corporation, Austin, TX, USA)	25
	PCR	(Hs00992441_m1) ABI-Prism 7300 Sequence Detector System (Applied Biosystems	7
IL-32	PCR	IL-32 (Hs00992441_m1), ABI-Prism 7300 Sequence Detector System	29
IL-32α	PCR	IL-32a (Hs04353657_gH), ABI-Prism 7300 Sequence Detector System	29
IL-32β	PCR	IL-32b (Hs04353658_gH), ABI-Prism 7300 Sequence Detector System	29
IL-32g	PCR	IL-32c (Hs04353656_g1), ABI-Prism 7300 Sequence Detector System	29
IL-32d	PCR	IL-32d (Hs04353659_gH), ABI-Prism 7300 Sequence Detector System	29
IL-36α	ELISA	Rabbit polyclonal anti-IL-36a (C-terminal; ab180909), from Abcam, Cambridge, U.K. at 1 : 500 dilution.	3
	ELISA	IL-36a AF1078, RnD	28
IL-36β	ELISA	Rabbit polyclonal anti- IL-36b (C-terminal; ab180890) from Abcam, Cambridge, U.K. at 1 : 500 dilution.	3
	ELISA	AF1099, RnD	28
IL-36g	ELISA	Mouse monoclonal anti-IL-36c ab156783; (Abcam, Cambridge, U.K.) at 1 : 500 dilution.	3
	ELISA	AF2320, RnD	28
IL-36RA	ELISA	Rabbit polyclonal from Abcam, Cambridge, U.K. at 1 : 500 dilution.	3
	ELISA	AF1275, RnD	28
IL-37	ELISA	Rabbit polyclonal Abcam, Cambridge, U.K. at 1 : 500 dilution.	3
IL-38	ELISA	Rabbit polyclonal Abcam, Cambridge, U.K. at 1 : 500 dilution.	3
TNF-α	ELISA	TNF-alpha: 559071 mABG 1:10 mouse antihuman	24
	ELISA/ ECL	ELISA (Quantikine; R&D Systems) or Luminex assay (Millipore, Billerica, MA).	4
	ECL	Meso Scale Discovery electrochemiluminescent assay (MSD, Meso Scale Diagnostics, Rockville, MD MSD V-Plex cytokine panel 1 and the V-plex proinflammatory panel 1	6
	ELISA	Cytokines were measured in duplicate by ELISA (R&D Minneap- olis, USA).	5
	PCR	Taqman gene expression assays (Applied Biosystems) on a Roche Light Cycler	31
	ECL	(CBA Human Inflammation kit and CBA Human TH1⁄TH2 Cytokine kit; BD Biosciences, Franklin Lakes, NJ, U.S.A.) Analyssi: FACSCalibur (BD Biosciences)	26
TNF-β	ECL	Meso Scale Discovery electrochemiluminescent assay (MSD, Meso Scale Diagnostics, Rockville, MD MSD V-Plex cytokine panel 1 and the V-plex proinflammatory panel 1	6
	ECL	(CBA Human Inflammation kit and CBA Human TH1⁄TH2 Cytokine kit; BD Biosciences, Franklin Lakes, NJ, U.S.A.) Analyssi: FACSCalibur (BD Biosciences)	26
sTNFR1	ECL	xMAP technology (Luminex Corporation, Austin, TX, USA)	8
	ECL	xMAP technology (Luminex Corporation, Austin, TX, USA)	25
	ECL	(CBA Human Inflammation kit and CBA Human TH1⁄TH2 Cytokine kit; BD Biosciences, Franklin Lakes, NJ, U.S.A.) Analyssi: FACSCalibur (BD Biosciences)	26
sTNFR2	ECL	xMAP technology (Luminex Corporation, Austin, TX, USA)	8
	ECL	xMAP technology (Luminex Corporation, Austin, TX, USA)	25
	ECL	(CBA Human Inflammation kit and CBA Human TH1⁄TH2 Cytokine kit; BD Biosciences, Franklin Lakes, NJ, U.S.A.) Analyssi: FACSCalibur (BD Biosciences)	26
hBD1	ELISA/ ECL	ELISA (Quantikine; R&D Systems) or Luminex assay (Millipore, Billerica, MA).	4
hBD2	ELISA/ ECL	ELISA (Quantikine; R&D Systems) or Luminex assay (Millipore, Billerica, MA).	4
hBD3	ELISA	ELISA 1 : 400; rabbit antihuman	24
	ELISA/ ECL	ELISA (Quantikine; R&D Systems) or Luminex assay (Millipore, Billerica, MA).	4
S100A7	ELISA	Psoriasin HL15-4 mAbG 1:20,000 mouse antihuman	24
	ELISA/ ECL	ELISA (Quantikine; R&D Systems) or Luminex assay (Millipore, Billerica, MA).	4
S100A8	ELISA/ ECL	ELISA (Quantikine; R&D Systems) or Luminex assay (Millipore, Billerica, MA).	4
	ELISA	S100A8 and S100A9 (monospecific affinity-purified rabbit antisera to S100A8 and to S100A9	30
S100A9	ELISA/ ECL	ELISA (Quantikine; R&D Systems) or Luminex assay (Millipore, Billerica, MA).	4
	ELISA	S100A8 and S100A9 (monospecific affinity-purified rabbit antisera to S100A8 and to S100A9	30
LL37	ELISA	Cathelicidin ab64892 pAbG 1:1000 rabbit antihuman	24
Lyzozyme	ELISA	Lysozyme A0099 pAbG 1:100 rabbit antihuman	24
MIF	ELISA	MIF MAB289 mABG 1:100 mouse antihuman	24
αMSH	ELISA	alpha MSH M09393 mABG 1:500 rabbit antihuman	24
MHC1	ELISA	MHC1 W6/32 mABG 1:50 mouse antihuman	24
RNase7	ELISA/ ECL	ELISA (Quantikine; R&D Systems) or Luminex assay (Millipore, Billerica, MA).	4
IP10	ELISA/ ECL	ELISA (Quantikine; R&D Systems) or Luminex assay (Millipore, Billerica, MA).	4
CCL3	ECL	(CBA Human Inflammation kit and CBA Human TH1⁄TH2 Cytokine kit; BD Biosciences, Franklin Lakes, NJ, U.S.A.) Analyssi: FACSCalibur (BD Biosciences)	26
CCL5	ELISA/ ECL	ELISA (Quantikine; R&D Systems) or Luminex assay (Millipore, Billerica, MA).	4
	ECL	(CBA Human Inflammation kit and CBA Human TH1⁄TH2 Cytokine kit; BD Biosciences, Franklin Lakes, NJ, U.S.A.) Analyssi: FACSCalibur (BD Biosciences)	26
CCL20	ELISA/ ECL	ELISA (Quantikine; R&D Systems) or Luminex assay (Millipore, Billerica, MA).	4
CCL27	ELISA/ ECL	ELISA (Quantikine; R&D Systems) or Luminex assay (Millipore, Billerica, MA).	4
CRP	ECL	xMAP luminex Luminex Corporation, Austin, TX, USA	8
	ECL	xMAP luminex Luminex Corporation, Austin, TX, USA	25
ESR	ECL	xMAP luminex Luminex Corporation, Austin, TX, USA	8
	ECL	xMAP luminex Luminex Corporation, Austin, TX, USA	25
IFNg	PCR	(Hs00174143_m1), ABI-Prism 7300 Sequence Detector System (Applied Biosystems)	7
	ELISA	ELISA kits from Sanquin (Amsterdam, The Nether- lands)	9
	ECL	Meso Scale Discovery electrochemiluminescent assay (MSD, Meso Scale Diagnostics, Rockville, MD MSD V-Plex cytokine panel 1 and the V-plex proinflammatory panel 1	6
	ELISA/ ECL	ELISA (Quantikine; R&D Systems) or Luminex assay (Millipore, Billerica, MA).	4
GMCSF	ECL	Meso Scale Discovery electrochemiluminescent assay (MSD, Meso Scale Diagnostics, Rockville, MD MSD V-Plex cytokine panel 1 and the V-plex proinflammatory panel 1	6
	ELISA	Quantibody Human Inflammation array 3 (RayBiotech Inc., Norcross, GA, U.S.A.).	26
VEGF	ECL	Meso Scale Discovery electrochemiluminescent assay (MSD, Meso Scale Diagnostics, Rockville, MD MSD V-Plex cytokine panel 1 and the V-plex proinflammatory panel 1	6
sVEGFR1	ELISA	Quantikine enzyme-linked immunosorbent assay (ELISA) systems from Bio-Techne	27
Caspase 1	ELISA	Kelly *et al.* Caspase-1 fluorochrome inhibitor of caspases (FLICA) (ImmunoChemistry Technologies, Bloomington, MN, U.S.A.	30
NLRP3	PCR	Kelly IL10, IL17A, IL1 **Β**, IL18 and NLRP3 was performed with predesigned Taqman gene expression assays (Applied Biosystems) on a Roche Light Cycler (Pleasanton, CA, U.S.A.)	30
CAMP	PCR	(Hs00189038_m1) ABI-Prism 7300 Sequence Detector System (Applied Biosystems)	7
Uteroglob	ELISA	Quantikine enzyme-linked immunosorbent assay (ELISA) systems from Bio-Techne	27
Cystatin C	ELISA	Quantikine enzyme-linked immunosorbent assay (ELISA) systems from Bio-Techne	27
LCN2	ELISA	Quantikine enzyme-linked immunosorbent assay (ELISA) systems from Bio-Techne	27
BD2	ELISA	Quantikine enzyme-linked immunosorbent assay (ELISA) systems from Bio-Techne	27
MMP2	ELISA	Quantikine enzyme-linked immunosorbent assay (ELISA) systems from Bio-Techne	27
BLC	ECL	(CBA Human Inflammation kit and CBA Human TH1⁄TH2 Cytokine kit; BD Biosciences, Franklin Lakes, NJ, U.S.A.) Analyssi: FACSCalibur (BD Biosciences)	26
ICAM-1	ECL	(CBA Human Inflammation kit and CBA Human TH1⁄TH2 Cytokine kit; BD Biosciences, Franklin Lakes, NJ, U.S.A.) Analyssi: FACSCalibur (BD Biosciences)	26
Eotaxin	ECL	(CBA Human Inflammation kit and CBA Human TH1⁄TH2 Cytokine kit; BD Biosciences, Franklin Lakes, NJ, U.S.A.) Analyssi: FACSCalibur (BD Biosciences)	26
Eotaxin2	ECL	(CBA Human Inflammation kit and CBA Human TH1⁄TH2 Cytokine kit; BD Biosciences, Franklin Lakes, NJ, U.S.A.) Analyssi: FACSCalibur (BD Biosciences)	26
CXCL6	ELISA	Quantikine enzyme-linked immunosorbent assay (ELISA) systems from Bio-Techne	27
CXCL9	ECL	(CBA Human Inflammation kit and CBA Human TH1⁄TH2 Cytokine kit; BD Biosciences, Franklin Lakes, NJ, U.S.A.) Analyssi: FACSCalibur (BD Biosciences)	26
CXCL11	ELISA	Quantikine enzyme-linked immunosorbent assay (ELISA) systems from Bio-Techne	27
CX3CL1	ELISA	Quantikine enzyme-linked immunosorbent assay (ELISA) systems from Bio-Techne	27
I-309	ECL	(CBA Human Inflammation kit and CBA Human TH1⁄TH2 Cytokine kit; BD Biosciences, Franklin Lakes, NJ, U.S.A.) Analyssi: FACSCalibur (BD Biosciences)	26
MCP1	ECL	(CBA Human Inflammation kit and CBA Human TH1⁄TH2 Cytokine kit; BD Biosciences, Franklin Lakes, NJ, U.S.A.) Analyssi: FACSCalibur (BD Biosciences)	26
M-CSF	ECL	(CBA Human Inflammation kit and CBA Human TH1⁄TH2 Cytokine kit; BD Biosciences, Franklin Lakes, NJ, U.S.A.) Analyssi: FACSCalibur (BD Biosciences)	26
MIP1b	ECL	(CBA Human Inflammation kit and CBA Human TH1⁄TH2 Cytokine kit; BD Biosciences, Franklin Lakes, NJ, U.S.A.) Analyssi: FACSCalibur (BD Biosciences)	26
MIP1d	ECL	(CBA Human Inflammation kit and CBA Human TH1⁄TH2 Cytokine kit; BD Biosciences, Franklin Lakes, NJ, U.S.A.) Analyssi: FACSCalibur (BD Biosciences)	26
PDGF-BB	ECL	(CBA Human Inflammation kit and CBA Human TH1⁄TH2 Cytokine kit; BD Biosciences, Franklin Lakes, NJ, U.S.A.) Analyssi: FACSCalibur (BD Biosciences)	26
TIMP1	ECL	(CBA Human Inflammation kit and CBA Human TH1⁄TH2 Cytokine kit; BD Biosciences, Franklin Lakes, NJ, U.S.A.) Analyssi: FACSCalibur (BD Biosciences)	26
TIMP2	ECL	(CBA Human Inflammation kit and CBA Human TH1⁄TH2 Cytokine kit; BD Biosciences, Franklin Lakes, NJ, U.S.A.) Analyssi: FACSCalibur (BD Biosciences)	26

Table 4: Antibodies Used for Identification of Cytokines in Studies Included in this Systematic Review. ECL: Electrochemicoluminescence

### Assessment of bias

Assessment of bias is presented in Table 6. Two of the 14 questions regarding participation rate and loss to follow up were considered not applicable. All included studies identified clear objectives and a clearly defined study population. No clear inclusion or exclusion criteria were specified for 17 of the 19 studies. Power estimation was made for one study ^33^, and recording of all exposures (disease activity, comorbidities etc) were made prior to assessment of the outcomes (cytokine levels). The timeframe of analysis was sufficient to identify an association, but only 10 of the 19 studies (52.6%) documented different levels of exposures (disease severity, metabolic comorbidities, family history etc). There were no serial measures of cytokine levels in the majority of studies. Only three studies ^5, 25, 33^, examining cytokine levels after monoclonal antibody administration has measurements at two distinct time points. Outcomes of interest (cytokine levels) were measured consistently within studies, however there was great variance in the methods of measurement and analysis between studies ( Table 5). No studies took into account known confounding variables into analysis of their results by stratification or regression analyses.

**Table 6.** Risk of bias across studies included in this review.

Study Reference	1. Was the research question or objective in this paper clearly stated?	2. Was the study population clearly specified and defined?	3. Was the participation rate of eligible persons at least 50%?	. Were all the subjects selected or recruited from the same or similar populations (including the same time period)? Were inclusion and exclusion criteria for being in the study prespecified and applied uniformly to all participants?	5. Was a sample size justification, power description, or variance and effect estimates provided?	6. For the analyses in this paper, were the exposure(s) of interest measured prior to the outcome(s) being measured?	7. Was the timeframe sufficient so that one could reasonably expect to see an association between exposure and outcome if it existed?	8. For exposures that can vary in amount or level, did the study examine different levels of the exposure as related to the outcome (e.g., categories of exposure, or exposure measured as continuous variable)?	9. Were the exposure measures (independent variables) clearly defined, valid, reliable, and implemented consistently across all study participants?	10. Was the exposure(s) assessed more than once over time?	11. Were the outcome measures (dependent variables) clearly defined, valid, reliable, and implemented consistently across all study participants?	12 Were the outcome assessors blinded to the exposure status of participants?	13. Was loss to follow- up after baseline 20% or less?	14. Were key potential confounding variables measured and adjusted statistically for their impact on the relationship between exposure(s) and outcome(s)?
Moran *et al.* ^2^	Y	Y	N/A	N	N	Y	Y	Y	Y	N	Y	NR	N/A	N
Emelianov *et al.* ^24^	Y	Y	N/A	N	N	Y	Y	N	Y	N	Y	NR	N/A	N
Hessam *et al.* ^3^	Y	Y	N/A	N	N	Y	Y	N	Y	N	Y	NR	N/A	N
Hotz *et al.* ^4^	Y	Y	N/A	N	N	Y	Y	Y	Y	N	Y	NR	N/A	N
Thomi *et al.* ^7^	Y	Y	N/A	N	N	Y	Y	Y	Y	N	Y	NR	N/A	N
Jimenez- Gallo *et al.* ^8^	Y	Y	N/A	N	N	Y	Y	Y	Y	N	Y	NR	N/A	N
Banerjee *et al.* ^6^	Y	Y	N/A	N	N	Y	Y	N	Y	N	Y	NR	N/A	N
Jimenez- Gallo *et al.* ^25^	Y	Y	N/A	Y	N	Y	Y	N	Y	N	Y	NR	N/A	N
Kanni *et al.* ^5^	Y	Y	N/A	N	N	Y	Y	Y	Y	Y	Y	NR	N/A	N
Kelly *et al.* ^31^	Y	Y	N/A	N	N	Y	Y	N	Y	N	Y	NR	N/A	N
Lima *et al.* ^30^	Y	Y	N/A	N	N	Y	Y	N	Y	N	Y	NR	N/A	N
Ten Oever *et al.* ^9^	Y	Y	N/A	N	N	Y	Y	N	Y	N	Y	NR	N/A	N
Schlapbach *et al.* ^32^	Y	Y	N/A	N	N	Y	Y	Y	Y	N	Y	NR	N/A	N
Thomi *et al.* ^29^	Y	Y	N/A	N	N	Y	Y	Y	Y	N	Y	NR	N/A	N
Thomi *et al.* ^28^	Y	Y	N/A	N	N	Y	Y	Y	Y	N	Y	NR	N/A	N
Wolk *et al.* ^27^	Y	Y	N/A	N	N	Y	Y	N	Y	N	Y	NR	N/A	N
Van der Zee *et al.* ^26^	Y	Y	N/A	N	N	Y	Y	Y	Y	N	Y	NR	N/A	N
Kanni *et al.* ^33^	Y	Y	N/A	Y	Y	Y	Y	Y	Y	N	Y	NR	N/A	N

Key: Y = Yes; N= No, NR= Not Reported N/A = Not Applicable

### Assessment of heterogeneity

36 of the 81 identified cytokines or inflammatory proteins were assessed by more than 1 study. 23 of those cytokines had raw data available. No studies had sufficient measures of spread in order to calculate I ^2^measure of heterogeneity and so chi-squared statistic was used as an alternate marker of heterogeneity ( Table 7) along with a funnel plot ( Figure 3). In total, 18 individual cytokines (78.2%) were found to demonstrate heterogeneity. Only eight cytokines (Serum IL-10, Lesional IL-1α, IL-12p70, hBD1, hBD2, hBD3, S100A9 and GMCSF) illustrated homogeneity. Due to this high level of heterogeneity and concerns regarding the methodological quality of included studies, meta-analysis was not deemed appropriate to perform.

**Table 7.** Table of heterogeneity of cytokine studies by chi-squared tests for homogeneity.

Cytokine	Chi Squared	P
**IL1a Lesional**	**0.3525**	**p=0.552705**
IL1b Lesional	153.5947	p<0.00001
IL4 Lesional	4.3992	P=0.035955
IL5 Lesional	15.1692	P=0.000098
IL6 Lesional	461.9724	P<0.00001
IL8 Lesion	846.6251	P<0.0001
IL8 Serum	94.4212	P<0.0001
IL10 Lesion	90.3211	P<0.0001
**IL10 Serum**	**0.1595**	**P=0.689624**
IL12p40 Lesional	4.9618	P=0.025913
**IL12p70 Lesional**	**2.2116**	**P=0.136973**
IL13 Lesional	5.4163	P=0.019949
IL15 Lesional	39.2837	P<0.00001
IL16 Lesional	126.1959	P<0.00001
IL17A Lesional	22.6668	P<0.00001
IL17A Serum	19.1621	P=0.000012
TNFa Lesional	6.9761	P=0.030561
TNFb Lesional	7.4004	P=0.006521
**hBD1 Lesional**	**2.3317**	**P=0.311656**
**hBD2 Lesional**	**0.6488**	**P=0.722954**
**hBD3 Lesional**	**1.0314**	**P=0.597084**
S100A7 Lesional	621.2537	P<0.00001
S100A8 Lesional	19.6371	P=0.000054
**S100A9 Lesional**	**1.27**	**P=0.529927**
RNAse 7	6.7263	P=0.034626
**GMCSF Lesional**	**1.9405**	**P=0.163611**

## Discussion

The overall quality of reporting in the identified studies was low with little consistency between methodologies and cytokines examined. There was also great variability in the ages, genders, comorbidities, associated conditions and treatments of the patients included in these studies. This was again reflected in the high number of cytokines with statistical heterogeneity ( Table 7). The studies presenting conflicting data are often those studies with lower numbers of patients as well as lack of matched controls and/or lack of stratification by treatment. Meta-analysis using individual patient data would be required in order to account for these factors and re-assess the relationship between lesional and control cytokine levels.

In assessing the relationship between lesional and peri-lesional tissue, it has been demonstrated by many authors that different cytokines are present in peri-lesional tissue as opposed to lesional tissue. The definition of peri-lesional tissue is fairly consistent in the studies examined being 2cm from an active HS nodule on unaffected skin. However, no studies reported ultrasound examination of the peri-lesional skin to ensure that subclinical extension of the adjacent nodule (either in the dermis or the subcutaneous tissue) was being inadvertently sampled. This is an important differentiation to make in terms of identifying the subclinical pathogenic processes that precipitate this disease.

The raw data collated illustrates a number of paradoxically elevated levels of control cytokines (IL-15, IL-16) ( Table 4). Many of these control readings lie near the lower detection limit of specific assays in individual papers, and thus the possibility of erroneously elevated control readings cannot be excluded. The wide interquartile ranges of studies which did report individual patient data ^7^, suggest that analyzing aggregate data is not optimal and is prone to misrepresentation of the relationship between clinical disease, comorbidities and cytokine levels. Furthermore, high levels of heterogeneity within the measurements of individual cytokines suggest that examination of and correction for other variables or confounders is required.

### Methodological quality

Regarding methods of cytokine analysis, a number of authors have identified variability in cytokine levels measured with different forms of multiplex assays as well as traditional ELISA methods ^35– 39^. Different methods of cytokine analysis are known to be prone to variability, with some cytokines more sensitive than others. For example, IFN- **γ** and IL-1β were overestimated compared with ELISA methods ^37^, whilst IL-6 levels were underestimated ^37^. IL-6 levels when compared across four different multiplex assays showed significant variation in detectable range, accuracy and responsiveness ^36^. The correlation of TNF-α between ELISA and Multiplex assays was also poor (r=0.31) ^36^. Issues also exist with minimum detectable levels of cytokines with specific bead-based arrays ^36^ As an example, minimal detectable dose readings reported for IL-12p70 using some multiplex arrays ^39^ are higher than the levels reported in lesional HS samples ^6^. Therefore, whilst the general trends in the level of consistently elevated or suppressed cytokines in HS are reliable, the quantification of individual cytokines as well as the relationship between comorbidities and cytokine levels requires further research with consistent, reliable and accurate methodologies in order to further dissect the inflammatory cascade in this disease.

### Keratinocyte mediated inflammatory pathways

**Figure 2.**
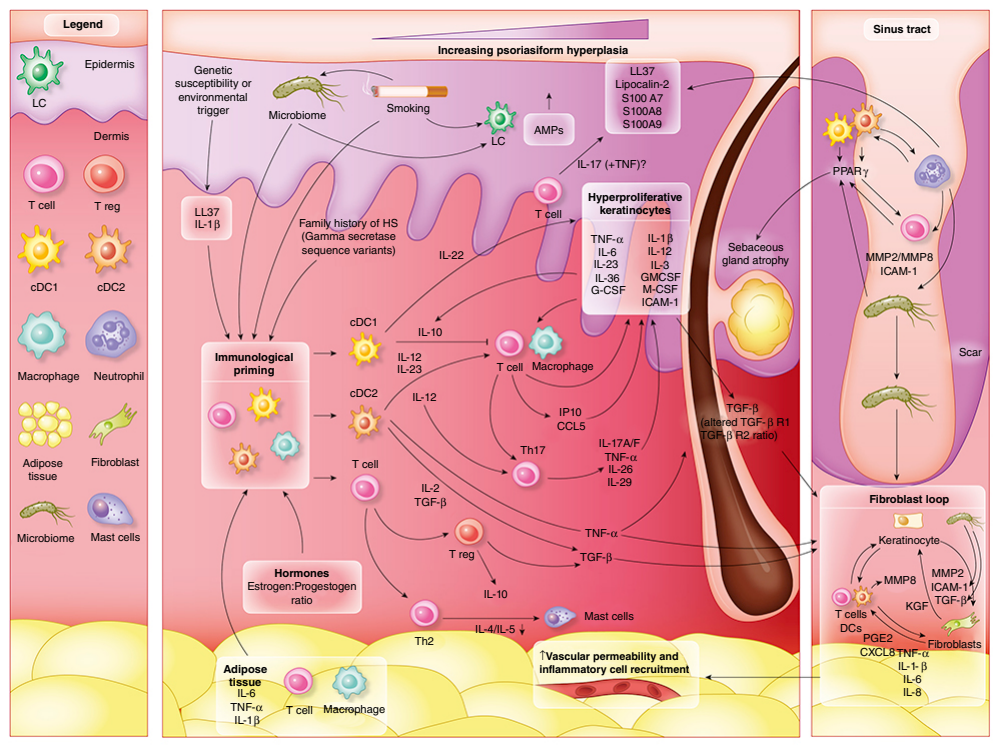
Inflammatory pathways in hidradenitis suppurativa, a schematic representation of the results identified in this systematic review. Immunological ‘priming’ occurs due to the contribution of adipose tissue, genetic susceptibility, smoking-related inflammatory mediators and obesity related pro-inflammatory signals and the composition of the microbiome. Increased activity of cDC1, cDC2 and T cells lead to both keratinocyte hyperplasia via the actions of IL-12 and IL-23, as well as a Th17 predominant immune response. Alterations of antimicrobial peptides (AMP’s) also occur throughout the epidermis. The dermal inflammation interacting with the hyperplastic epidermis result leads to a self-perpetuating inflammatory feed forward mechanism mediated by IL-36, Il-1B and TNF-a. The development of scarring and sinus tracts is associated with MMP2, ICAM-1 and TGF-Beta, with possible augmentation of ICAM-1 and TGF-B signaling via specific components of the microbiome. TNF-a, PGE2 and CXCL2 then lead to additional feed forward mechanisms perpetuating the inflammatory cycle.

The majority of elevated cytokines and inflammatory proteins identified in lesional skin of HS (TNF-α, IL-1β, IL-6, IL-8, IL-11, IL-23, IL-17A, IL-33, IL-36, LL-37, S100A7, S100A8, S100A9, GM-CSF, TGF-β, hBD2, hBD3, CCL3, CXCL9, CXCL11, PDGF, CCL5, CCL-20, MIF, GM-CSF and LCN2) are those known to be produced by keratinocytes, as well as perpetuating a self-amplification pathway ^34^ ( Figure 2). Additionally T-cells produce IL-17A, IL-17F, IL-26, IL-29, and IFN-γ; dendritic cells produce IL-12, IL-23 and possibly IL-39; neutrophils produce S100A8 and S100A9 (calgranulin); and innate lymphoid cells also contribute IFN-γ, IL-17A and IL-17F. This inflammatory model has been well documented and explored in both psoriasis and atopic dermatitis ^34, 40^. The psoriasiform epidermal hyperplasia seen in HS (mediated by IL-17 and maintained by IL-23-mediated T _h_17 stimulation) ^34^ reflects this common inflammatory pathway.

**Figure 3.**
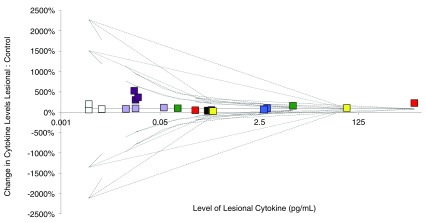
Funnel plot of selected cytokine in lesional and control samples of hidradenitis suppurativa. IL-1a = Red, IL-10 = Blue, IL-12p70 = Green, hBD1 = Purple, hBD2 = light purple, hBD3 = Black, S100A9 = White, GMCSF = Yellow.

The other elevated non-keratinocyte produced cytokines in HS (IL-4, IL-5, IL-10, IL-16, IL-17A, IL-22, IL-32, IL-36, hBD1), are produced by a combination of dendritic cells, monocytes, neutrophils and CD4+ T cells. IL-4 and IL-5 as key cytokines in the T _h_2 axis are consistent with the findings of Mast cells in HS ^41^, as well as the pruritus, which is frequently reported by patients. IL-10 in HS is produced by Treg cells ^2^ (although dendritic cells may also be a source), and whilst quantitatively the IL-10 signal appears paradoxically elevated, it can be explained by the up-regulation of T cells including Treg cells, which although significantly elevated from baseline, are not elevated enough in comparison to T _H_17/IL-17/IL-22 signal to counteract this strong pro-inflammatory cascade ^2^. Further exploration of these cytokines may reveal the initial trigger(s) of the inflammatory cascade in HS, or correlations with known pro-inflammatory comorbidities.

### Insights into pathogenesis of HS

In light of investigations in psoriasis and atopic dermatitis, the role of dendritic cells in HS needs to be clarified, as dendritic cell influx has been reported in histological studies ^41, 42^, and they may contribute to the high IL-10 and IL-15 levels reported. IL-32 is a second cytokine produced by dendritic cells, but has only been reported in one study ^29^. Further research into the functional role of IL-32 in the activity of dendritic cells in HS would be of value. The role of IL-20, IL-22, IL-24 and IL-26 needs further clarification. IL-19, TSLP and CCL17 (TARC) have not yet been examined in HS and this is required in order to further explore the role of dendritic cell, monocyte and T cell activation and migration in this disease.

It is well established that smoking, obesity and diabetes are strongly associated with HS ^13– 19, 42, 43^. The immunological effects of smoking include increase in number and responsiveness of dendritic cells, altered function of Treg cells and activation of Th17 pathways ^44^, whilst obesity and diabetes can result in production of IL-1β, IL-6 and TNF-α through activated macrophages in adipose tissue ^45, 46^. These potential mechanistic pathways (which may prime or contribute towards inflammation in HS) require validation in functional studies. However, if they are a significant contributor to inflammation, the presence or absence of these comorbidities need to be considered in future cytokine studies as confounding variables in order to identify significant biochemical markers independent of these other pro-inflammatory states that reflect the pathogenesis of HS.

The role of the microbiome ^42, 43^ in stimulating chronic inflammation has parallels in diabetes ^47^ and colonic inflammation ^48^ and the presence of *Porphyromonas* and *Peptoniphilus* species has been associated with a subpopulation of patients with HS ^42^. *Porphyromonas* has been associated with systemic inflammation and atherosclerosis through aberrant toll-like-receptor 4 signalling ^48^ and is not part of the natural cutaneous flora ^43^. Altered cutaneous and gastrointestinal microbiome can also act via microbiome metabolites (including lipopolysaccharides, short chain fatty acids and bile salts) ^49^ through stimulation of myeloid dendritic cells via G Protein Coupled Receptors (including GPR41, GPR43 and GPR109A) ^49, 50^. The microbiome may be implicated as a trigger factor for the initial inflammatory cascade in HS in a proportion of patients. Similarly, the presence of genetic polymorphisms as reported in HS ^51^ have the potential to up-regulate inflammatory activity through shedding of IL-6R, IL-15R, TNF-α ^52^ as well as up-regulating the response of dendritic cells to LPS stimulation via ADAM17 (which has been demonstrated to be elevated in a published gene expression study of HS) ^53^. These pathways may be involved prior to the activation of keratinocyte-mediated inflammation, and hence, may reveal novel targets for new interventions to control the disease prior to the onset of destructive inflammation.

### Limitations, interpretation and generalisability

The limitations to this study include the high degree of methodological variability ( Table 5) and high impact of bias ( Table 6) within the included studies. The lack of individual patient data has also prevented any further analysis into the contribution of comorbidities such as smoking and obesity to variable levels of cytokines in lesional tissue and/or serum. This, along with the high level of heterogeneity in many cytokines ( Table 7), has resulted in analyses of the collated data being limited to descriptive analyses only and limited the generalisability of results.

## Conclusions

Through this review we have catalogued the various cytokines that have been reported as elevated in lesional, peri-lesional tissue, serum or exudate of HS patients. We have also identified those cytokines with inconsistent results and identified methodological factors that may explain variability in findings. We have identified a number of missing links in disease pathogenesis with respect to cytokine actions and pathways that must be addressed in future work. Areas for further investigation include the role of dendritic cells in HS, the contribution of obesity, smoking, diabetes and the microbiome to cytokine profiles in HS, and examining the natural history of the disease through longitudinal measurements of cytokines over time.

## Data availability

All data underlying the results are available as part of the article and no additional source data are required.

### Extended data

 OSF: Extend data. Data Collection Sheet Cytokine. Review HS. https://doi.org/10.17605/OSF.IO/N2E7A ^22^

License: CC0 1.0 Universal

### Reporting guidelines

OSF: PRISMA checklist for ‘A systematic review and critical evaluation of inflammatory cytokine associations in hidradenitis suppurativa’. https://doi.org/10.17605/OSF.IO/N2E7A ^22^

License: CC0 1.0 Universal
