# Supplementary material for: A systematic review and critical evaluation of inflammatory cytokine associations in hidradenitis suppurativa
Source: F1000Res. 2018 Dec 13;7:1930. [Version 1] doi: 10.12688/f1000research.17267.1 (PMC6392156; doi:10.12688/f1000research.17267.1)
Supplement: Peer review history

# Peer review: Reviewer response for version 1

**DOI:** 10.5256/f1000research.18879.r43994

**Published:** 2019-03-04

**Authors:** Evangelos Giamarellos-Bourboulis

**Affiliations:** 1 Attikon University Hospital, Athens, Greece; 2 National and Kapodistrian University of Athens, Athens, Greece

This is a long time needed review trying to shed light in the pathogenesis of hidradenitis suppurativa (HS). My concerns are coming from the biggest hurdle the authors had to overcome from the very beginning of their attempt i.e. the great heterogeneity of the existing evidence. Due to this, I find over-exaggerated the conducted approach to set-up a mechanistic interpretation for the disease. I believe that the heterogeneity is so vast that it is almost impossible to suggest the pathways implicated in the pathogenesis of HS. To this end, I suggest that the mechanistic parts are omitted and Figure 2 as well.

I have read this submission. I believe that I have an appropriate level of expertise to confirm that it is of an acceptable scientific standard, however I have significant reservations, as outlined above.

# Peer review: Reviewer response for version 1

**DOI:** 10.5256/f1000research.18879.r43995

**Published:** 2019-02-25

**Authors:** Aude Nassif

**Affiliations:** 1 Institut Pasteur, Medical Center, Paris, France

This very instructive study aims at analyzing previous cytokine studies in HS patients, in skin tissue, blood, serum and exudates, to assess relevancy and reliability of these studies.

 The authors have performed an extensive work, methods seem perfectly appropriate. The authors are very critical and rigorous in their approach, looking for confounding factors, which is highly desired.

 The authors could also mention that genetic heterogeneity may play a role in the diversity of results and encourage using similar phenotypes for future studies.

 This analysis brings up a very important and honest contribution to the current knowledge in cytokines involved in HS and therefore deserves indexing.

I have read this submission. I believe that I have an appropriate level of expertise to confirm that it is of an acceptable scientific standard.

# Peer review: Reviewer response for version 1

**DOI:** 10.5256/f1000research.18879.r43493

**Published:** 2019-02-07

**Authors:** Barbara Horváth, Lisette Prens

**Affiliations:** 1 Department of Dermatology, University of Groningen, Groningen, The Netherlands

Thank you for the opportunity to review this manuscript and congratulations to the authors for their great efforts in putting this systematic review together. Research on the role of cytokines in HS is important, as it may lead to new targets for therapy and a better understanding of the pathophysiology of HS.

**Summary**

 This systematic review focused on collecting all data published on cytokine studies in tissue, blood, serum and exudate in hidradenitis suppurativa. 81 discrete cytokines were examined in HS patients (n=564) and control patients (n=198) in 19 studies. Methodology varied greatly among studies, which were generally of low quality. When measuring levels of cytokines, substantial variance was found and the majority of cytokines showed heterogeneity. IL-17 signalling appeared to be a significant component. Suggestions for further research were discussed.

**Questions**

**Are the rationale for, and objectives of, the Systematic Review clearly stated?**

 Yes.

**Are sufficient details of the methods and analysis provided to allow replication by others?**

 Yes. However, I wonder why the term ‘hidradenitis suppurativa’ is not in the search strategy and ‘hidradenitidis suppurative’ is? ‘Hidradenitidis’ is not an existing word, as far as I know and will not provide any search results. Please adjust.

**Is the statistical analysis and its interpretation appropriate?**

 Yes, as far as I can judge as a non-statistician. The analyses used are ones I have little experience with myself. I’ll refrain from commenting on this section.

**Are the conclusions drawn adequately supported by the results presented in the review?**

 Partly. The last conclusion ‘examining the natural history of the disease through longitudinal measurements of cytokines over time’ is not discussed anywhere else in this article. First, I suggest changing ‘history’ to ‘course’. Moreover, I am wondering, how the authors propose to do this. Monitoring the natural course of the disease, would mean patients cannot receive any treatment for their HS, during this proposed study. Depending on how long the natural course is meant to be monitored, I don’t think it is ethical to withhold patients from treatment.

 Please elaborate on this conclusion with a specific proposal or otherwise rephrase or maybe leave out this conclusion.

**Other comments**

 Page 9 last paragraph/Page 28 – 1 ^st^ paragraph: You state that ‘psoriasiform epidermal hyperplasia is seen in HS’. Please provide a reference for this statement. The reference provided only references to the pathway likely responsible for this in psoriasis.

 Page 28 – 4 ^th^ paragraph: ‘These potential mechanistic pathways (which may prime or contribute towards inflammation in HS) require validation in functional studies.’ Could you please provide an example on how such a functional study should be designed to produce reliable results?

 Table 4: the abbreviation ‘Lpa’ is not clarified in the key section of the table. Does ‘Le’ (page 11, IL-1a, first row) mean lesion exudate? 

 Table 6: the number four of question four is missing in the top row of the table on both pages (24-25). Please insert.

We have read this submission. We believe that we have an appropriate level of expertise to confirm that it is of an acceptable scientific standard.
